# A rare case of mesentric ischaemia post laparoscopic cholecystectomy

**DOI:** 10.1093/jscr/rjaf090

**Published:** 2025-02-28

**Authors:** Ved Prakash, Mirza Faraz, Abhishek Sharma, Anirudh Agrawal

**Affiliations:** Asian Institute of Medical Sciences, Faridabad, Haryana 121001, India; Asian Institute of Medical Sciences, Faridabad, Haryana 121001, India; Department of General Surgery, Asian Institute of Medical Sciences, Faridabad, Haryana 121001, India; Department of General Surgery, Asian Institute of Medical Sciences, Faridabad, Haryana 121001, India

**Keywords:** intestinal ischaemia, cholecystectomy, pneumo-peritoneum, thrombosis, co-morbidities

## Abstract

Laparoscopic cholecystectomy (LC) has become the standard treatment for gallstones. Current experience indicates that in more than 95% of cases, the procedure is safe and can be performed without complications with mortality rate of 0.2%. Death following this surgery is devastating to people all around the world as it is the safest and most common surgery done world-wide. Intestinal ischaemia is a rarely reported complication following LC. We describe a case of massive small intestinal necrosis 2 days following this procedure. After excluding all the known causes, we determined the cause to be splanchnic hypoperfusion, likely due to the physiologic changes induced by the pneumoperitoneum and elevated intra-abdominal pressure, necessary to conduct the surgery, among all the cases reported of mesenteric ischaemia have comorbidities and, in our case, the patient is having no comorbidities.

## Introduction

Intestinal ischemia following laparoscopic cholecystectomy (LC) is a rarely reported complication, but one that often results in a fatal outcome. Several case reports in the past decade have served to illustrate that ischaemia in this context has diverse causes [1–3]. Most have been due to thrombosis of the superior mesenteric artery. In this report, we describe a case of fatal intestinal ischaemia following LC of which splanchnic hypoperfusion during pneumoperitoneum was the most likely cause and, in our case, the patient was having no co-morbidities.

## Case report

A 79-year-old female with symptomatic gallstone disease and upper abdominal pain presented to emergency department, with no h/o any cardiovascular disease and no other co-morbidities; the diagnosis was confirmed to be cholelithiasis with choledocholithiasis.

**Figure 1:**
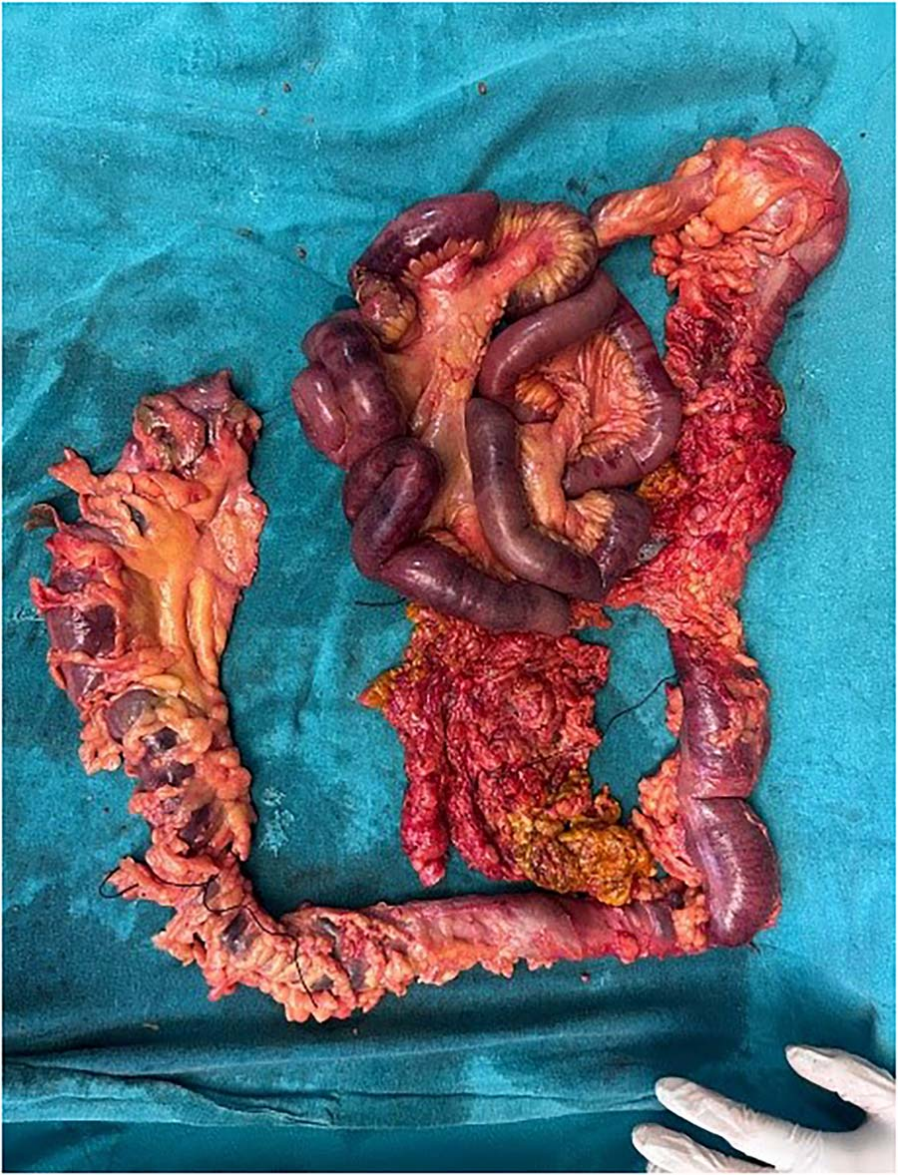
Resected intestinal segment

**Figure 2:**
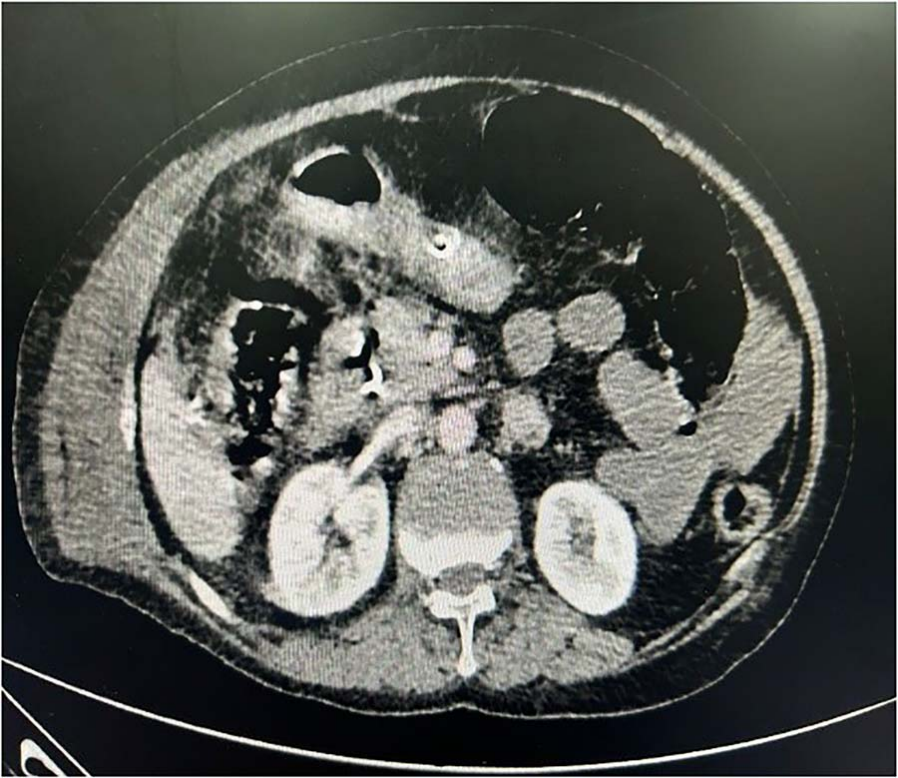
CECT whole abdomen of the patient post laparoscopic cholecystectomy

The patient was planned for surgery LC: the procedure lasted for 80 min and was performed under low flow anesthesia using sevo-flurane as the principal agent. Carbon dioxide pneumoperitoneum was maintained with intra-abdominal pressure of 12–14 mmHg. Intra-op omentum was adherent with frozen calot triangle. LC was performed without complication with the placement of abdominal drain in the sub-hepatic space. Post-operative, the patient was clinically stable, on pod-2, the patient had diffuse abdominal pain with abdominal distension, CT was done (Fig. 1) and it was s/o mild collection in anterior abdominal wall, we suspected bile leak, patient was planned for emergency laparotomy, intra op cystic duct stump leak was found and gangrenous bowel was found from mid jejunum to sigmoid colon, ileo-colic resection and end ileostomy done (Fig. 2), the remaining bowel also appears to be dusky and was not showing any signs of good vascularity. The patient condition was deteriorating after surgery and the ileostomy site showed ischaemic changes with severe sepsis and DIC, and the patient died on pod-5. Histopathological examination of the resected bowel showed features of venous infarction with patchy mucosal necrosis and oedema. No granulomas were present.

## Discussion

Intestinal ischaemia following LC is a rare but with devastating complication. A literature review using PubMed and pertinent search terms revealed eight cases before the present report (Table 1).

**Table 1:** Reported cases of intestinal ischaemia after laparoscopic cholecystectomy

**Case**	**Age/sex**	**Duration (minutes)**	**IAP (mmHg)**	**Onset of symptoms**	**Distribution of ischaemia**	**Vascular thrombosis?**	**Outcome**
Paul (1994)	68/M	85	15	Day 4	Ileum and right colon		Death
Jaffe (1994)	76/F	70		Day 3	Entire small bowel and right colon	Yes: SMA	Death
Thiele (1994)	87/M			Day 4		Yes: SMA	Death
Dwerryhouse (1995)	36/M	50	15	30 hours	Ileum	No	Recovery
Schorr (1995)	62/F	40		Day 3	Small bowel and right colon	Yes: SMA	Death
Klugewitz (1998)	41/M			24 hours	Colon	Yes: IMV	Recovery
Andrei (1999)	72/F	50	15	Day 8	Entire small bowel		Death
Leduc (2005)	57/F	120	15	Day 3	Entire small bowel	No	Death
Bhanumati (2024)	79/F	80	15	Day 2	Mid ileum to sigmoid colon	No	Death

In most of the cases reported, the patient has various co-morbidities leading to either thrombosis of SMA and IMA; in our case, the patient did not have co-morbidities and CECT did not show any atheromatous plaque and there were no risk factors pertaining to mesenteric ischaemia, nor any coagulation abnormalities.

The chief hypothesis put forward to explain bowel ischaemia following LC is the intra-abdominal hypertension created by the pneumoperitoneum, which has been showed to reduce mesenteric perfusion [4]. Diminished cardiac output and mesenteric venous outflow produced by elevated IAP may be other contributing factors [4]. In addition, carbon dioxide pneumoperitoneum may lead to mesenteric vasoconstriction by its local absorption [5]. Although these physiologic changes create a theoretical risk of compromised intestinal blood flow in all patients, in the vast majority, they are well tolerated with no clinical consequences.

The patient we describe had no clinical evidence of significant cardiovascular disease. The absence of atheromatosis of the major intraabdominal and mesenteric vessels, and the absence of venous thromboembolism, both excluded by detailed examination at the time of biopsy, eliminates these as causes of ischaemia. Likewise, a faulty operative technique or intestinal obstruction due to external compression or herniation may also be eliminated. We conclude that the massive intestinal ischaemia in this patient is most likely due to splanchnic hypo perfusion, the physiologic basis of which is described above [7].

Concerning preventive measures, Paul *et al*. [1] suggested that in patients in whom pre-existing impairment of splanchnic blood flow is suspected, it may be wise to use intermittent decompression of gas during pneumoperitoneum or an alternative technique.

Junghans *et al*. [6] proposed that the head-up position should be avoided, if possible, to prevent further reduction in the cardiac output. The most logical approach would be, however, to perform the procedure using the lowest possible insufflation pressure. In addition to the preservation of mesenteric blood flow, such practice has been shown to result in better postoperative pulmonary function and less pain [8]. We conclude that surgeons performing laparoscopic procedures should be familiar with the variety of physiological changes that occur after the induction of carbon dioxide pneumoperitoneum. Mesenteric ischaemia should be considered in the differential diagnosis in patients developing nonspecific abdominal symptoms after laparoscopic procedures, and patients with advancing age even with no significant co-morbidities should be screened to rule out mesenteric ischaemia.
